# Novel foci of *Dermacentor reticulatus* ticks infected with *Babesia canis* and *Babesia caballi* in the Netherlands and in Belgium

**DOI:** 10.1186/s13071-015-0841-2

**Published:** 2015-04-17

**Authors:** Frans Jongejan, Moniek Ringenier, Michael Putting, Laura Berger, Stefan Burgers, Reinier Kortekaas, Jesse Lenssen, Marleen van Roessel, Michiel Wijnveld, Maxime Madder

**Affiliations:** Utrecht Centre for Tick-borne Diseases (UCTD), FAO Reference Centre for Ticks and Tick-borne Diseases, Faculty of Veterinary Medicine, Utrecht University, Yalelaan 1, 3584 CL Utrecht, The Netherlands; Current address: Institute for Hygiene and Applied Immunology, Center for Pathophysiology, Infectiology and Immunology, Medical University of Vienna, Kinderspitalgasse 15, 1090 Vienna, Austria; Unit of Veterinary Entomology, Department of Biomedical Sciences, Institute of Tropical Medicine, Nationalestraat 155, B2000 Antwerp, Belgium; Department of Veterinary Tropical Diseases, Faculty of Veterinary Science, University of Pretoria, Private Bag X04, Pretoria, Onderstepoort 0110 South Africa

**Keywords:** Ticks, *Dermacentor reticulatus*, Netherlands, Belgium, *Babesia canis*, *Babesia caballi*

## Abstract

**Background:**

Autochthonous populations of *Dermacentor reticulatus* ticks in the Netherlands were discovered after fatal cases of babesiosis occurred in resident dogs in 2004. The presence of *D. reticulatus* in the Netherlands has also linked with the emergence of piroplasmosis in the resident horse population. The aim of this study was to put together results of continued surveillance of field sites and hosts for this tick in the Netherlands and also in Belgium and determine their infection status for *Babesia* and *Theileria* species.

**Methods:**

Ticks were collected from the vegetation at 11 locations between 2011 and 2013. *D. reticulatus* ticks were also collected from different hosts between 2007 and 2013. Ticks were screened by PCR and reverse line blot (RLB)*.*

**Results:**

A total of 1368 *D. reticulatus* ticks were collected from 4 previously known field locations and from 5 new locations in the Netherlands and from 2 sites in Belgium (one old and one new location). A total of 855 ticks collected from 8 locations in the Netherlands and 2 locations in Belgium were tested. Fourteen ticks (1,64%) collected at 4 field locations (Dintelse Gorzen, Rozenburg, Slikken van de Heen and St. Philipsland) were positive for *Babesia canis*, whereas two ticks were positive for *Babesia caballi*, one tick in the Dintelse Gorzen in the Netherlands and one tick was found positive in De Panne in Belgium.

A further 1092 *D. reticulatus* ticks were collected between 2007 and 2013 from 40 dogs (132 ticks), two ticks from two humans, 51 ticks from 15 horses, two ticks from two cats, one tick from a roe deer, whereas most ticks (904) were collected from cattle (n = 25). Ticks were found throughout the year on dogs in nearly all provinces of the Netherlands. None of the ticks collected from these hosts were infected.

**Conclusions:**

*D. reticulatus* is continuing its spread into novel areas. The finding that some autochthonous ticks are infected with *B. canis* and *B. caballi* poses a threat to the resident dog and horse population and justifies year-round tick control measures.

## Background

The ornate dog tick, *Dermacentor reticulatus*, is a Palearctic species with a highly focal distribution pattern [1,2]. It occurs in isolated pockets from south-western England in the west to Central Asia until the Yenisei river basin in Siberia in the east [3-7].

*D. reticulatus* is an important vector of a number of protozoan tick-borne pathogens, in particular *Babesia canis*, the cause of canine babesiosis, and *Babesia caballi* and *Theileria equi,* which are causative agents of equine piroplasmosis [8]. Recently, *Babesia microti* has also been identified in questing adult *D. reticulatus* ticks, which suggests a role in the transmission of human babesiosis [9].

Moreover, *D. reticulatus,* collected from various locations in the Lublin region of eastern Poland, were found infected with tick-borne encephalitis (TBE) virus [10]. In addition, *D. reticulatus* collected by flagging from the vegetation in urban areas (Warsaw) as well as in national parks in the North-Eastern part of Poland were also infected with TBE virus [11].

Spotted Fever Group Rickettsiae (SFGR) causing Siberian tick typhus (*Rickettsia sibirica*)*,* and human rickettsiosis (*Rickettsia slovaca*) have also been found in *Dermacentor* ticks [8]. Moreover, *Rickettsia raoultii*, has been detected in *Dermacentor* ticks by PCR in many European countries, including the Netherlands [12]. Both *R. slovaca* and *R. raoultii* are associated with human cases of tick-borne lymphadenopathy (TIBOLA) or *Dermacentor*-borne necrosis erythema and lymphadenopathy (DEBONEL) [13]. Interestingly, *R. raoultii* has recently been isolated and maintained in tick cell cultures initiated from eggs derived from a colony of Dutch *D. reticulatus* ticks [14].

*Anaplasma phagocytophilum,* causing tick-borne fever in ruminants as well as granulocytic anaplasmosis in humans, horses, dogs and cats, has recently also been detected in questing *D. reticulatus* ticks collected from the vegetation in Lithuania [15] and also in questing ticks in the Chernobyl exclusion zone [16]. Moreover, in Belgium, *A. phagocytophilum* was found in ticks collected from a red deer [17]. Finally, the tick has proven vector competence for *Anaplasma marginale* [18] and *A.marginale-* infected field ticks were recently reported from France [19].

Surveillance for field populations of *D. reticulatus* ticks started in 2004 in the Netherlands, when outbreaks of autochthonous canine babesiosis occurred affecting 23 dogs [20]. Nineteen animals recovered after treatment, whereas four dogs died. Adult *D. reticulatus* ticks were collected from three of these dogs. Although at the time of the outbreaks no *D. reticulatus* ticks were found in the vegetation in the walking area of these dogs, the presence of autochthonous populations of *D. reticulatus* ticks in the Netherlands was confirmed and reported in 2007 [12].

In addition to the emergence of babesiosis in dogs, evidence has recently been presented that equine piroplasmosis has emerged in the South-West of the Netherlands [21]. Two acute clinical *Theileria equi* cases and subclinical *Babesia caballi* infections were diagnosed in resident horses with a sero-prevalence of 1.3% among 300 horses in the South-West of the country [21]. As a result, the local *D. reticulatus* tick population has also been incriminated with autochthonous transmission of *Babesia caballi* and *Theileria equi*.

By launching the so-called “Tickbusters” survey, initially by the Royal Netherlands Society of Veterinary Medicine (KNMvD) in 2005, veterinarians throughout the Netherlands started to submit ticks collected in their practices from companion animals to the Utrecht Centre for Tick-borne Diseases (UCTD) [22]. Through interviews with persons who submitted *D. reticulatus* ticks, we were able to discover eight locations where local populations of *D. reticulatus* ticks were present in the vegetation [12]. After a decade of Tickbusters surveying, wherein more than 63,000 ticks were received, we now report additional field locations for *D. reticulatus* as well as ticks removed from dogs, horses, cattle, deer and humans between 2007 and 2013.

Similar developments have been reported in Belgium, where *D. reticulatus* tick populations were found at four distinct locations in the vegetation [5].

In this study *D. reticulatus* ticks collected from the vegetation as well as from a range of different hosts were examined for the presence of *Babesia* and *Theileria* parasites using PCR combined with reverse line blot (RLB) hybridization.

## Methods

### Tick collections

Ticks were submitted to our Reference Centre by veterinarians who removed the ticks from dogs or cats presented by pet owners in their clinics. The following data were collected: host, location, date of tick collection, engorgement status of female ticks and information whether the tick-infested pet had been outside the Netherlands during the period of tick attachment. All ticks were identified to species level with stage and sex recorded using descriptions published in a guide to the identification of ticks of domestic animal in the Mediterranean region [23]. All tick submissions were assigned a unique database number and stored in 70% ethanol until further use. When *D. reticulatus* ticks were found on an animal, the veterinarian or owner was contacted in order to find out more about the actual location where the animal may have acquired the tick. By doing so, novel field locations of resident populations of *D. reticulatus* were revealed. These areas were subsequently monitored for the presence of ticks by flagging, which is considered more effective than dragging for collecting adult ticks [24]. Additional *D. reticulatus* ticks were received that had been collected from horses, cats, cattle, roe deer and humans. Efforts were made to track the ticks to where they had been picked up by these hosts in order to locate additional field sites.

### DNA extraction, PCR amplification and reverse line blot hybridization

DNA was extracted from individual ticks using the NucleoSpin® Tissue Kit (Macherey-Nagel, Germany). Ticks were disrupted in lysis buffer using the TissueLyser LT (Qiagen, The Netherlands) with 5 mm stainless steel beads according to the instructions of the manufacturer. All genomic DNA was stored at – 20°C until used for PCR amplification. *Babesia/Theileria* PCR was performed using primers RLB-F2 (5′-GACACAGGGAGGTAGTGACAAG-3′) and RLB-R2 (biotin-5′-CTAAGAATTTCACCTCTGACAGT-3′) to amplify a fragment of 460–540 bp from the 18SrRNA gene spanning the V4 region [25]. Reactions were performed in 25 μl volumes in PCR Buffer, 200 μM of dNTP, 400 μM of each primer, 0.125 μl of Phire Hot Start II Polymerase (Fisher Scientific, The Netherlands) and 2.5 μl of genomic DNA. Further PCR conditions based on a touch-down assay were recently described by [26]. Positive and negative controls were included in each run.

Reverse line blot (RLB) hybridization was performed according to methods originally published by Gubbels et al. (1999) [27], modified by Nijhof et al. (2003) [28] and updated by Giangaspero et al. (2015) [26]. In brief, oligonucleotide probes containing an N-terminal C6 amino linker (Eurogentec, The Netherlands) were covalently linked to the RLB membrane (Biodyne C blotting membrane; Pall Biosupport, Ann Arbor, MI, USA) using the following procedure. Membranes were activated in a freshly prepared 10-ml solution of 16% 1-ethyl 3-(3-dimethylaminopropyl) carbodiimide HCl (Sigma, St. Louis, MO), rinsed, and then placed in an MN45 miniblotter (Immunetics, Cambridge, MA) and residual liquid aspirated. Oligonucleotide probes (400 pmol/150 μl in 500 mM NaHCO3 solution (pH 8.4) were linked to the membrane by loading into the lanes of the miniblotter. After aspiration of the oligonucleotide probe solutions, the membrane was washed and inactivated in a 100 ml freshly prepared 100 mM NaOH solution at room temperature under gentle shaking. The RLB membrane was hybridized with the PCR products and further developed as described elsewhere [27].

## Results

### Tick collections

**Figure 1:**
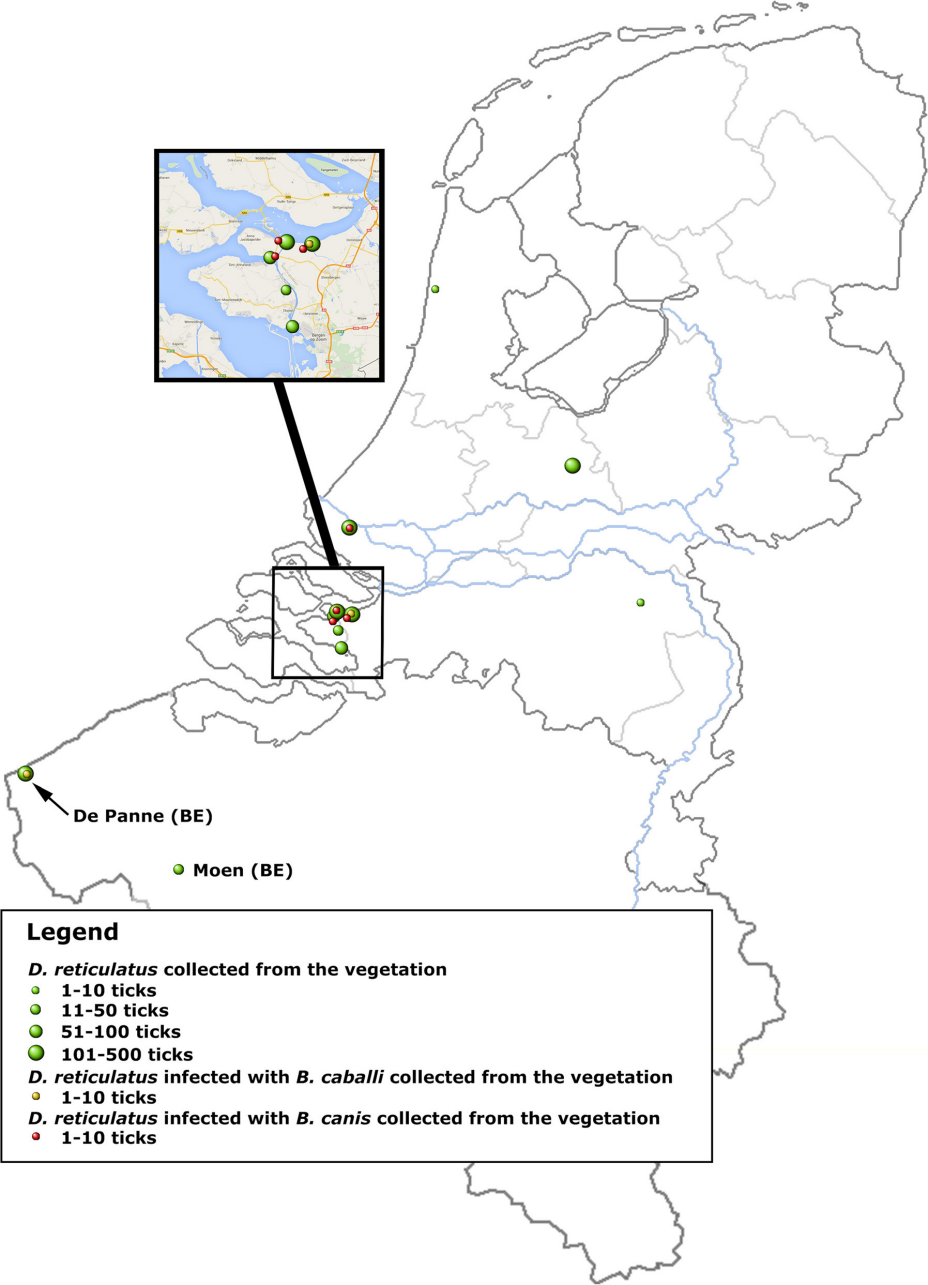
Map of the Netherlands and Belgium showing all sites where *Dermacentor reticulatus* ticks were collected from the vegetation.

*D. reticulatus* ticks were collected by flagging the vegetation in four previously known field sites and in five novel locations in the Netherlands and from two sites in Belgium (one known and one novel location) (Figure 1). A total of 1368 (583 males and 785 female ticks) were collected in 11 sites from 2011 to 2013 (Table 1).

**Table 1:** **Eleven locations in the Netherlands (n = 9) and Belgium (n = 2) where** ***Dermacentor reticulatus ticks*** **were collected from the vegetation**

**Location**	**Date (Month-Year)**	**Male**	**Female**	**Total ticks per collection**	**Total ticks per location**
De Maashorst	April-12	0	2	2	2
De Panne*	October-11	97	111	208	287
	March-12	16	23	39	
	April-12	9	13	22	
	December-13	7	11	18	
Dintelse Gorzen	November-11	20	20	40	236
	March-12	51	59	110	
	April-12	5	7	12	
	September-12	1	5	6	
	December-13	25	43	68	
Egmond aan Zee	March-12	0	1	1	4
	October-12	1	0	1	
	December-13	1	1	2	
Utrechtse Heuvelrug	March-12	18	35	53	208
	April-12	52	99	151	
	October-12	3	1	4	
Moen*	April-12	10	13	23	23
Oud-Vossemeer	September-12	9	2	11	29
	December-13	5	13	18	
Rozenburg	January-11	19	33	52	220
	February-11	27	38	65	
	March-11	8	11	19	
	September-11	20	20	40	
	March-12	0	1	1	
	December-13	21	22	43	
Slikken van de Heen	March-12	40	49	89	228
	April-12	25	30	55	
	September-12	20	16	36	
	November-13	20	28	48	
St. Philipsland	March-12	23	32	55	63
	November-13	2	6	8	
Tholen	September-12	11	6	17	68
	October-12	0	11	11	
	November-13	17	23	40	
Total		**583**	**785**	**1368**	

*These locations are situated in Belgium.

**Figure 2:**
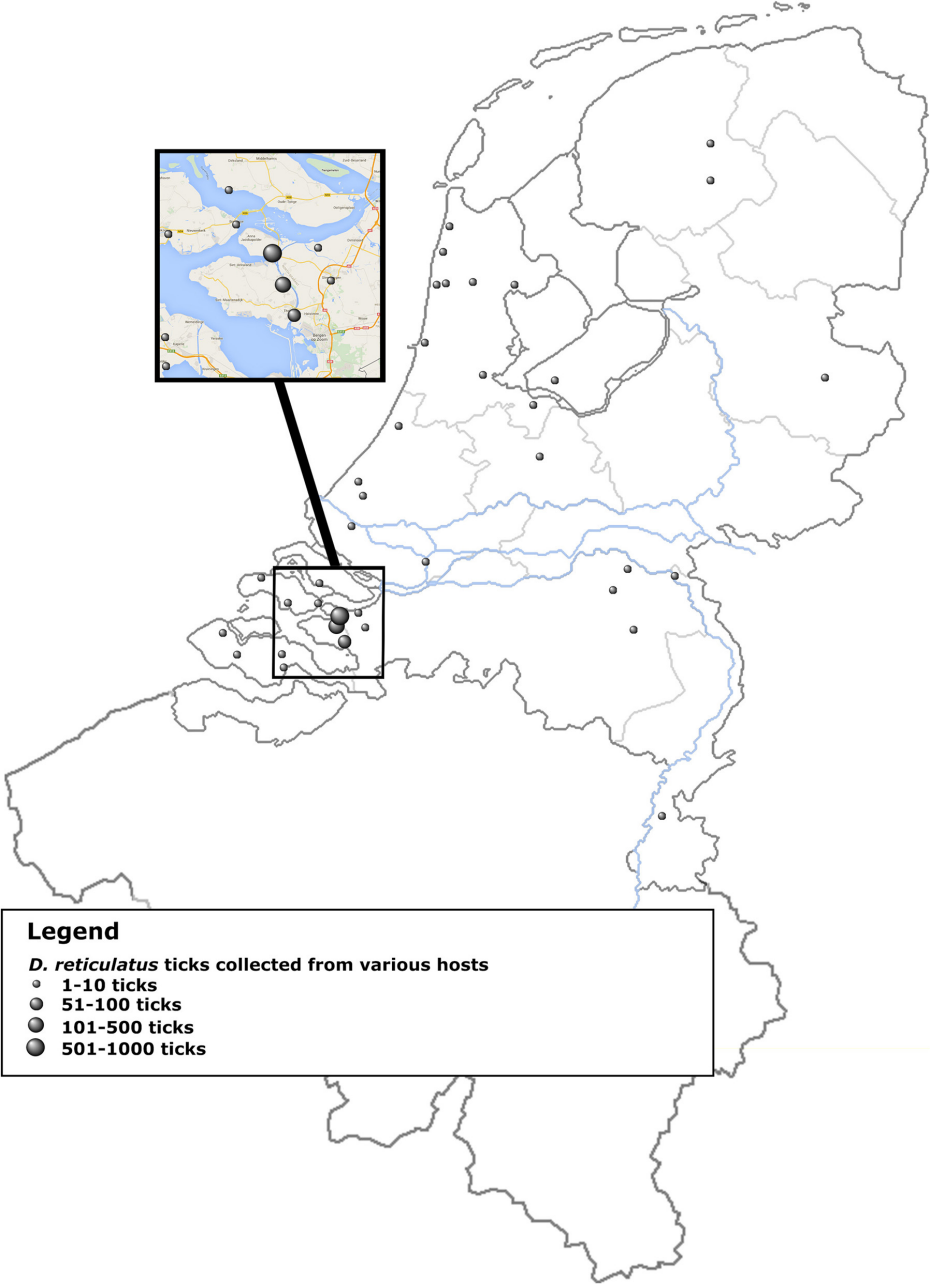
Map of the Netherlands and Belgium showing all sites where *Dermacentor reticulatus* ticks were collected from a range of different hosts.

From various hosts, a total of 1092 ticks (681 males and 411 females) were collected between 2007 and 2013: 132 ticks from 40 dogs, two ticks recovered from two humans, 51 ticks collected from 15 horses, two ticks from two cats, one tick from a roe deer and 904 ticks were collected from cattle (n = 25) (Figure 2; Table 2).

**Table 2:** ***Dermacentor reticulatus*** **ticks collected from various hosts in the Netherlands**

**Location**	**Date (Month-Year)**	**Host (number)**	**Province**	**Number of ticks (male/female)**
Steenbergen	January-07	Human	Drenthe	1 (1/-)
	October-11	Dog, horse	Drenthe	2 (1/1)
Almere-stad	May-08	Dog	Flevoland	1 (−/1)
Drachten	July-08	Dog	Friesland	1 (−/1)
Gorredijk	September-10	Dog	Friesland	1 (−/1)
Geleen	June-09	Dog	Limburg	1 (−/1)
Middelaar	August-10	Dog	Limburg	1 (1/-)
De Maashorst	May-09	Horses (2)	Noord-Brabant	2 (−/2)
Dintelse Gorzen	November-08	Horse	Noord-Brabant	1 (1/-)
	May-09	Roedeer		1 (−/1)
Gemert	September-08	Dog	Noord-Brabant	1 (1/-)
Reek	May-08	Dog	Noord-Brabant	1 (−/1)
Amsterdam	September-09	Dog	Noord-Holland	1 (1/-)
Bergen	December-13	Human	Noord-Holland	1 (−/1)
Bussum	May-09	Dog	Noord-Holland	4 (−/4)
Egmond aan den Hoef	September-07	Dog	Noord-Holland	1 (1/-)
Egmond aan Zee	October-12	Dog	Noord-Holland	1 (1/-)
	April-13	Dog		1 (1/-)
	October-13	Dog		1 (1/-)
	December-13	Dog		1 (1/-)
Heerhugowaard	November-09	Dog	Noord-Holland	1 (−/1)
Hoorn	September-10	Dog	Noord-Holland	1 (−/1)
Sint Maartenszee	February-09	Dog (2)	Noord-Holland	6 (−/6)
Wijk aan Zee	January-13	Dog	Noord-Holland	1 (1/-)
Almelo	April-07	Dog	Overijssel	1 (−/1)
De Bilt	March-12	Dog	Utrecht	1 (−/1)
Bruinisse	February-07	Dogs (2)	Zeeland	2 (1/1)
Grevelingen	October-13	Dog	Zeeland	1 (1/-)
’s Heer Arendskerke	March-07	Dog	Zeeland	1 (1/-)
Hoedekenskerke	May-2012	Dog	Zeeland	1 (−/1)
Middelburg	September-07	Dog	Zeeland	1 (1/-)
Oud-Vossemeer	November-09	Cattle	Zeeland	121 (40/81)
	September-12	Horse		5 (1/4)
	October-12	Horse		31 (18/13)
Renesse	May-10	Horse	Zeeland	7 (−/7)
Serooskerke	March-12	Horse	Zeeland	3 (3/-)
Tholen	September-12	Dogs (2)	Zeeland	5 (3/2)
	October-12	Dog		3 (2/1)
	January-13	Dogs (3)		61 (20/41)
	April-13	Dogs (3)		24 (7/17)
Zierikzee	April-07	Dog	Zeeland	1 (1/-)
	September-10	Cat		1 (1/-)
Den Haag	March-10	Dog	Zuid-Holland	1 (−/1)
Noordwijk	September-09	Dog	Zuid-Holland	1 (−/1)
Rijswijk	March-09	Cat	Zuid-Holland	1 (1/-)
Rozenburg	February-11	Dog	Zuid-Holland	1 (−/1)
Zwijndrecht	November-12	Dog	Zuid-Holland	1 (−/1)
Sint Philipsland	September-08	Cattle (25)	Zeeland	9 (5/4)
	November-08			297 (191/106)
	November-09			128 (74/54)
	November −11			40(1/39)
	November −12			309 (297/12)
**Total**				**1092 (681/411)**

### Pathogen detection

A total of 855 ticks collected from 8 locations in the Netherlands and 2 locations in Belgium were screened by PCR/RLB for the presence of *Babesia* and *Theileria* species (Table 3). Fourteen ticks (1.64%) collected in 4 different field locations (Dintelse Gorzen, Rozenburg, Slikken van de Heen and St. Philipsland) were positive for *B. canis*, whereas two ticks were positive for *B. caballi*; one was found in the vegetation in the Dintelse Gorzen in the Netherlands and one positive tick found in de Panne located in Belgium at the coast near the French border (Figure 1).

**Table 3:** **Pathogens detected in** ***Dermacentor reticulatus*** **ticks from the vegetation by PCR/RLB**

**Location**	**Total tested**	***B. caballi***	***B. canis***
De Maashorst	Not done	*-*	*-*
De Panne (BE)	266	1	Neg
Dintelse Gorzen	110	1	1
Egmond aan Zee	3	Neg	Neg
Utrechtse Heuvelrug	40	Neg	Neg
Moen (BE)	23	Neg	Neg
Oud-Vossemeer	27	Neg	Neg
Rozenburg	80	Neg	10
Slikken van de Heen	80	Neg	2
St. Philipsland	48	Neg	1
Tholen	56	Neg	Neg
**Total**	**855**	**2(0,23%)**	**14(1,64%)**

None of the ticks collected from dogs, cats, horses, cattle or humans were infected with any known *Babesia* or *Theileria* species.

## Discussion

Significant numbers of adult *D. reticulatus* ticks (n = 1368) were collected at nine locations in the Netherlands and at two locations in Belgium between 2011 and 2013 in autumn to early spring (September-April), but not in summer (Table 1). Most locations were found through the ongoing “Tickbusters” survey [22], although three locations (De Panne, Utrechtse Heuvelrug and Egmond aan Zee) were posted on flora and fauna observation data collection sites (www.waarneming.nl and www.waarnemingen.be). *D. reticulatus* was found to be abundant in freshwater tidal marches, such as Dintelse Gorzen (n = 236), Oud-Vossemeer (n = 29), Slikken van de Heen (n = 228), St. Philipsland (n = 63) and Tholen (n = 68), all situated in close proximity to waterways in the south-western part of the Netherlands (Figure 1). These locations are characterized by a high soil moisture and shelter against desiccation, which makes them suitable for tick survival, whereas the presence of populations of cattle, sheep, horses and even roe deer provide ample opportunity for adult ticks to feed on. Although no larvae or nymphs of this tick were collected, it can be assumed that immature ticks are being maintained in these locations on rodents during the summer months as is known to occur in other areas, e.g. France or Poland [7,29].

Several locations (Dintelse Gorzen, Slikken van de Heen and St. Philipsland) had been visited before and appear to sustain permanent populations of *D. reticulatus* since 2005 [12]. New locations within the same area in the South-West, and of similar ecological texture, are reported here (Oud-Vossemeer and Tholen) (Figure 1; Table 1).

Other locations where lower numbers of *D. reticulatus* had been reported previously include a deciduous forest (De Maashorst) (n = 12) and a dune valley (St. Maartenszee) (n = 11) [12]. In the current survey, only 2 ticks were found at De Maashorst, whereas 4 ticks were collected at Egmond aan Zee, a location near St. Maartenszee, both situated in a typical dune landscape with similar ecological characteristics near the cost in the north-western part of the country. Although deciduous forests and dune valleys appear to be less favourable areas for *D. reticulatus* to survive, a large number of ticks (n = 208) were found in a novel location situated in a deciduous forest (Utrechtse Heuvelrug) in the central part of the Netherlands. This is a recreational area characterized by a fragmented habitat consisting of deciduous forest, small lakes and heathland sustaining flocks of sheep. Moreover, whereas few ticks were found in the dune valleys in the Netherlands (Egmond aan Zee), large numbers of ticks (n = 287) were collected in Belgium in De Panne, a conservation area consisting of dune valleys close to the sea near the French border (Figure 1; Table 1). In this novel site in Belgium, ticks were most frequently found in high dry grass alongside a fence separating the dunes from fields where cattle and horses were grazing.

In the Netherlands, one further novel location was discovered near the city of Rotterdam (Rozenburg) (Figure 1) where a total of 220 ticks were collected and distributed over three different seasons (2011–2013). Rozenburg is characterized by a moist dune-like landscape, where cattle and horses graze freely and access to the public is made through dedicated fences.

In Belgium, Moen, a natural reserve of about 26 hectares along a canal accessible for recreational purposes, was revisited in 2012 and only 23 ticks were collected. *D. reticulatus* had been previously collected from the vegetation in Moen as well as in 3 other localities in Belgium as previously reported [5].

More than 60% of the ticks collected at the various field locations were screened by PCR/RLB (Table 3). Fourteen ticks (1.64%) collected in four locations (Dintelse Gorzen, Rozenburg, Slikken van de Heen and St. Philipsland) were positive for *B. canis*. Furthermore, two ticks were positive for *B. caballi*, one was found in the vegetation in the Dintelse Gorzen in the Netherlands and one tick was found in de Panne in Belgium. These are the first findings of indigenous field ticks infected with *B. canis* and *B. caballi* in the Netherlands and in Belgium, whereas ticks examined in previous surveys were negative for both parasites [5,12,30].

Dogs travelling to and from endemic areas in Europe have introduced infected *D. reticulatus* ticks. Once, introduced, *D. reticulatus* can sustain the *Babesia* infection for several generations. This can lead to the occurrence of autochthonous cases as previously reported in the Netherlands [20,31] and Belgium [32] and more recently also in Norway [33] and Switzerland [34]. The chance however for dogs to pick-up infected *D. reticulatus* ticks from infected field sites remains low because of the low infection rate. This may explain why additional clinical cases have not (yet) been reported in the Netherlands or in Belgium. However, infection rates of *B. canis* in *D. reticulatus* ticks vary from 0% in studies conducted for instance in Germany [35] to 2.3% (n = 1205) in south-western Slovakia to as high as 14.7% (n = 327) in eastern Slovakia [4].

The finding of *B. caballi*-infected ticks at the Dintelse Gorzen in the Netherlands and also in the Panne in Belgium provides additional evidence that equine piroplasmosis is transmitted by indigenous *D. reticulatus* ticks. Cases of *B. caballi* in horses reported in a survey conducted in the area in the south-western part of the Netherlands had been previously attributed to the presence of *D. reticulatus* [21]. Moreover, *D. reticulatus* ticks (n = 51) were found on horses (n = 15), which may have acquired them in or around two known locations (De Maashorst and Oud-Vossemeer) and in three novel locations (Table 2). Like *B. canis, B. caballi* can be maintained in several generations of *D. reticulatus* ticks. Although clinical cases have not been reported in Belgium, this does not mean that they have not occurred since there is no notification process in place. Treatment of clinical cases is probably the best approach, since prevention through tick control is limited because no product has been registered on the local markets.

Furthermore, *D. reticulatus* ticks (n = 132) were collected from 40 different dogs examined at 28 different locations throughout the Netherlands (Figure 2; Table 2). Importantly, dogs apparently did pick up ticks near or at locations known to harbour resident tick populations in the vegetation, demonstrated by the clustering around Egmond aan Zee, Oud-Vossemeer, Tholen and Rozenburg (Figure 1). Interestingly, ticks were found on dogs in all provinces in the Netherlands, except in Groningen and were found on dogs throughout the year, although very few ticks were seen during the summer months (Table 2; Figure 2). Often, ticks were engorged which demonstrates that dogs (and horses) contribute to the further dissemination of this tick. As a result, there were ticks found on hosts which could not be linked to any know field site, which indicates that most likely additional field locations in the Netherlands are infested with *D. reticulatus*. Adult activity of this tick throughout the year justifies year-round prophylaxis preferably using products that have been tested with respect to their ability to prevent transmission of *B. canis* [36-38].

A common denominator in all previous and novel locations is the presence of free-ranging populations of small as well as large ruminants. Also, most sites are located inside nature conservation areas with limited access to the public. However, most ticks were picked-up in patches alongside fences separating dog walking or horse trails from pastures and fields where ruminants are grazing. Ticks were also collected from animals grazing in some of the locations were *D. reticulatus* had been identified. Cattle in St. Philipsland, which were on pasture between April and November each year and grazing in typical freshwater tidal marches were found infested with large numbers of adult *D. reticulatus* ticks (n = 904 collected at four occasions between September 2008 and November 2012) (Table 2). This clearly demonstrates that this particular location provides ideal environmental conditions for permanent residency of *D. reticulatus* in the Netherlands.

The finding of a single tick on a roe deer in one of the know locations (Dintelse Gorzen) indicated that further surveillance for ticks on wildlife is required in order to determine the relative role of deer and other wildlife species in the dissemination of *D. reticulatus* in the Netherlands and in Belgium, where large wildlife populations are present. Finally, two ticks recovered from humans indicate that identification of ticks on humans is meaningful and could lead to zoonotic pathogen identification. Our findings are in agreement with the known host range for three-host *D. reticulatus* ticks, with immature ticks feeding on rodents and adult ticks feeding on a broad range of domestic and wild hosts, e.g. dogs, horses, cattle, sheep, deer, and swine) [2,7,23].

Clearly, changes in ecosystem management with consequent increased wildlife host abundance combined with grazing of large domestic ruminants in nature reserves has created favourable conditions to sustain *D. reticulatus* populations once introduced [39]. Although usually some *I. ricinus* ticks were also present on the vegetation or on hosts (cattle), *D. reticulatus* outnumbered *I. ricinus* at all locations that were visited (unpublished observations). Interesting, recent data show that *D. reticulatus* is the predominant tick species in the vegetation in selected areas of Slovakia [40]. However, widespread dissemination of *D. reticulatus*, into, for instance, deciduous forests, where *I. ricinus* predominates, is unlikely. In addition to the ecological requirements that need to be in place for the tick to survive, *D. reticulatus* nymphs do not feed as frequently as *I. ricinus* nymphs on birds, hence its horizontal dispersion is limited [23].

Finally, continued longitudinal surveillance is recommended including a broad molecular screening to encompass not only the confirmed *Babesia* and *Theileria* species, but also *Rickettsia* and *Anaplasma* species, and TBE virus since all these pathogens have been found in questing *D. reticulatus* ticks found elsewhere in Europe [11,13,15,19].

## Conclusion

This study showed that *D. reticulatus* ticks are slowly but steadily spreading within in the Netherlands and Belgium. Adult activity of this tick on dogs throughout the year justifies year-round prophylaxis. Continued surveillance is required to monitor the distribution of *D. reticulatus* and associated tick-borne diseases in the resident dog and horse population in the Netherlands and in Belgium.
